# Gleanings

**Published:** 1896-10

**Authors:** 


### GLEANINGS.

Eight cows died suddenly on one farm near Allentown recently, and an autopsy revealed only inflammatory patches of the mucous membrane of the paunches; all other organs were healthy.

David Waugh, Jr., of Western Pennsylvania, advises owners to avoid turning newly-castrated colts or horses into pastures containing rag-weeds, as the weeds prove very injurious and often fatal. He says it is bad policy to castrate horses in the country, where the blossoms are shed and afterward blown in all directions by the wind.

W. J. Waugh, V.S., once castrated two large draught-colts when horses were considered valuable property, and the colts were turned out in a rag weed pasture, where they soon sickened and died, notwithstanding all known measures were used to save them. The stern realities of practice in civil life ceased to have any charm for the young doctor thereafter, and he soon joined his elder brother in the United States Army.

An experiment recently reported to the French Academy of Science consisted of eight pigs which had been inoculated with tuberculosis; three of them were exposed for one hour daily to the x-rays for a period of eight weeks, and grew fat and thrived under the experiment, while the others developed abscesses and their condition became more unhealthy.

At time of calving, after severe pains, a mass containing the front members of the fœtus was found projecting from a cow; extending like an arch from the exit of the vagina was a round mass about the size of one's head and provided with a handle-like excrescence. This growth prevented the passage of the head of the calf. After removing this swelling calving followed without difficulty. After a microscopical examination the tumor was pronounced a lipoma. On calving some twenty months before a tumor as large as an egg had been noticed in the vagina of the same cow. Very often tumors are found in the vagina of cattle and obstruct calving. It is therefore advisable to remove all such with the knife as soon as they are observed, on account of the danger of infection when they are removed during calving.

